# Effects of continuous cropping of sweet potatoes on the bacterial community structure in rhizospheric soil

**DOI:** 10.1186/s12866-021-02120-6

**Published:** 2021-04-01

**Authors:** Zhiyuan Gao, Yaya Hu, Meikun Han, Junjie Xu, Xue Wang, Lanfu Liu, Zhonghou Tang, Weijing Jiao, Rong Jin, Ming Liu, Zhengjun Guan, Zhimin Ma

**Affiliations:** 1 Institute of Cereal and Oil Crops, Hebei Academy of Agriculture and Forestry Sciences, The Key Laboratory of Crop Genetics and Breeding of Hebei, Shijiazhuang, China; 2 Department of Life Science, Yuncheng University, Yuncheng, China; 3 Xuzhou Sweet Potato Research Center, Xuzhou Institute of Agricultural Sciences, Xuzhou, China; 4 Agricultural Product Quality Inspection Center of Shijiazhuang, Shijiazhuang, China

**Keywords:** Bacterial community, Continuous cropping, Rhizospheric soil, Sweet potato

## Abstract

**Background:**

Continuous cropping obstacles from sweet potatoes are widespread, which seriously reduce the yield and quality, causing certain economic losses. Bacteria of rhizospheric soil are the richest and are associated with obstacles to continuous cropping. However, few studies have examined how continuous sweet potato cropping affects the rhizospheric soil bacterial community structure.

**Results:**

In the study, the Illumina MiSeq method was used to explore the variations in

rhizospheric soil bacterial community structure of different sweet potato varieties after continuous cropping, as well as the correlation between soil characteristics and the bacterial community. The results showed that (1) the dominant bacterial phyla in rhizospheric soils from both Xushu 18 and Yizi 138 were *Proteobacteria*, *Acidobacteria*, and *Actinobacteria*. The most dominant genus was *Subgroup 6_norank*. The relative abundance of rhizospheric soil bacteria varied significantly between the two sweet potato varieties. (2) The richness and diversity indexes of bacteria were higher in Xushu 18 rhizospheric soil than in Yizi 138 soil after continuous cropping. Moreover, beneficial *Lysobacter* and *Bacillus* were more prevalent in Xushu 18, while Yizi 138 contained more harmful *Gemmatimonadetes*. (3) Soil pH decreased after continuous cropping, and redundancy analysis indicated that soil pH was significantly correlated with the bacterial community. Spearman’s rank correlation coefficient analysis demonstrated that pH was positively associated with *Planctomycetes* and *Acidobacteria*, but negatively associated with *Actinobacteria* and *Firmicutes*.

**Conclusions:**

After continuous cropping, the bacterial community structure and physicochemical properties of sweet potato rhizospheric soil were changed, and the changes from different sweet potato varieties were different. The contents of *Lysobacter* and *Bacillus* were higher in the sweet potato variety resistant to continuous cropping. It provides a basis for developing new microbial fertilizers for sweet potatoes to alleviate the continuous cropping obstacle.

## Background

Sweet potato [*Ipomoea batatas* (L.) Lam.] is a major food crop that is cultivated worldwide. It is a high-yield, highly efficient, and strongly adaptable crop that is resistant to drought and barrenness [1–3]. In addition, sweet potato is rich in nutrients and has cancer preventing properties, making it increasingly popular [4]. Therefore, sweet potato planting areas increase annually. In China, because of limited cultivatable land, sweet potatoes are grown continuously in the same fields. However, continuous cropping can lead to decreased yield and quality, severe plant death, and a decreased or non-existent harvest [5, 6]. In recent years, the phenomenon of sweet potato continuous cropping obstacles has become serious, and it has huge adverse impact on the sweet potato industry [7].

Continuous cropping obstacles have a certain relationship with soil enzyme activity, soil microbial community, and root exudates [8–10]. Soil microorganisms are the key factors related to changes in soil quality, fertility, and productivity [11–13]. The rhizosphere is the soil region near the plant roots, where interactions between soil microorganisms and plant root systems are very strong [14, 15]. Rhizospheric soil microorganisms are closely related to the absorption and transformation of soil nutrients. Therefore, their community structure is a major factor affecting plant growth, development, and health [16–19]. Many reports have indicated that continuous cropping changes the structure of rhizospheric soil microbes [20–23]. These changes have further led to severe continuous cropping obstacles [9]. Therefore, the relationship between continuous cropping and the soil microorganisms has become a research hotspot [7].

Bacteria are the most abundant in rhizospheric soil and are the important component [24, 25]. Increasing reports have noted that continuous cropping causes changes in rhizospheric soil bacterial community structure. Previous studies on konjac and *vanilla* revealed that continuous cropping changed bacterial communities, resulting in a decrease of beneficial bacteria and an increase of harmful bacteria [26, 27]. Moreover, the study found that the bacteria decreased after *Panax notoginseng* continuous cropping for 3 years. And the rhizospheric soil bacterial diversity of the healthy *Panax notoginseng* was greater than that of diseased strains. Canonical correspondence analysis found that P, pH, and soil organic matter had the greatest impacts on the bacterial community [28]. Furthermore, Na et al. [29] and Lei et al [30] revealed that the number of bacterial species and the α-diversities of the rhizospheric soil bacterial communities of *Lycium barbarum* L. and *Sophora flavescens* decreased significantly after continuous cropping.

The detrimental effects of continuous cropping on microbial community structures in rhizospheric soil have been demonstrated for a variety of different crops. However, studies focusing on the continuous cropping of sweet potatoes have mainly focused on the prevention and treatment of pests and diseases [31]. Accordingly, little is known about how this process affects the bacterial community structure and the physical and chemical properties (especially the medium and micro elements) in the rhizospheric soil of sweet potato. Various medium and micro elements are not only indispensable components of plants, but also participate in various metabolic reactions and act as catalysts for various enzymes [32]. Moreover, it remains unclear whether there are differences in the bacterial community structure of rhizospheric soil after the continuous cropping of different sweet potato cultivars. For the first time, we have used two sweet potato varieties and high-throughput techniques to study bacterial community structure changes of rhizospheric soil after continuous cropping, and analyzed correlations between soil characteristics and bacterial community changes after continuous cropping. It aimed to reveals how continuous cropping affects the rhizosphere soil bacterial community structures of two sweet potato varieties, and to provide insights into the application of biological control the continuous cropping obstacles of sweet potatoes.

## Results

### Physicochemical properties of sweet potato rhizospheric soil

Available Mn in the Xushu 18 (X18) and Yizi 138 (Y138) rhizospheric soils decreased by 32.68% and 31.14%, respectively, at the beginning of planting in 2016 than in 2015, and by 27.35% and 31.10% in the pre-harvest period (**Table** **1**). The soil pH of the X18 and Y138 were decreased by 2.72% and 3.11%, respectively, at the beginning of planting in 2016 than in 2015; changes in pH were not significant in the pre-harvest period. At the beginning of planting, available Ca in the X18 and Y138 rhizospheric soils increased by 29.80% and 38.97%, respectively; available Zn increased by 56.11% and 43.19%, respectively in 2016 than in 2015. And available Ca increased by 30.75% and 26.47%, respectively, while available Zn increased by 29.46% and 30.81%, respectively, in the pre-harvest period in 2016 than in 2015. Available Fe in the X18 and Y138 soils decreased by 18.61% and 17.08%, respectively, in the pre-harvest period in 2016 than in 2015, but was not significantly different at the beginning of planting. Available B in the X18 rhizospheric soil decreased by 20.63% at the beginning of planting, while the change in the Y138 soil was not significant.

**Table 1:** The physicochemical properties of different soil samples

Sample	Ca (g kg^-1^)	B (mg kg^-1^)	Fe (mg kg^-1^)	Mn (mg kg^-1^)	Zn (mg kg^-1^)	pH
X18-1	0.34±0.01c	0.77±0.07a	4.64±0.67c	2.60±0.28a	1.65±0.13d	8.57±0.06a
X18-2	0.35±0.01c	0.71±0.08ab	5.86±0.30a	2.54±0.06a	1.96±0.01c	8.60±0.00a
X18-3	0.45±0.01b	0.61±0.06b	4.78±0.16bc	1.75±0.07b	2.58±0.09a	8.33±0.06b
X18-4	0.45±0.00ab	0.63±0.05b	4.77±0.01bc	1.85±0.05b	2.54±0.02a	8.50±0.10a
Y138-1	0.34±0.02c	0.78±0.05a	4.52±0.24c	2.56±0.01a	1.73±0.06d	8.57±0.06a
Y138-2	0.36±0.01c	0.69±0.04ab	5.31±0.53ab	2.71±0.26a	1.66±0.17d	8.60±0.00a
Y138-3	0.47±0.02a	0.69±0.08ab	4.70±0.18bc	1.76±0.12b	2.48±0.00a	8.30±0.00b
Y138-4	0.46±0.01ab	0.72±0.09ab	4.41±0.03c	1.87±0.04b	2.17±0.03b	8.53±0.06a

Values are mean±standard deviation of triplicate determinations. Different letters in the same column indicate significant differences of the same sweet potato variaties at different sampling times at a level of P<0.05 using Duncan’s multiple range tests; Ca:available calcium; B: available boron; Fe: available iron; Mn : available Manganese; Zn: available zinc. X18: Xushu 18; Y138:Yizi 138; 1 and 2 represent sampling of early planting and early harvest in 2015; 3 and 4 represent sampling of early planting and early harvest in 2016, respectively

### Rhizospheric soil bacterial α-diversity

**Fig. 1:**
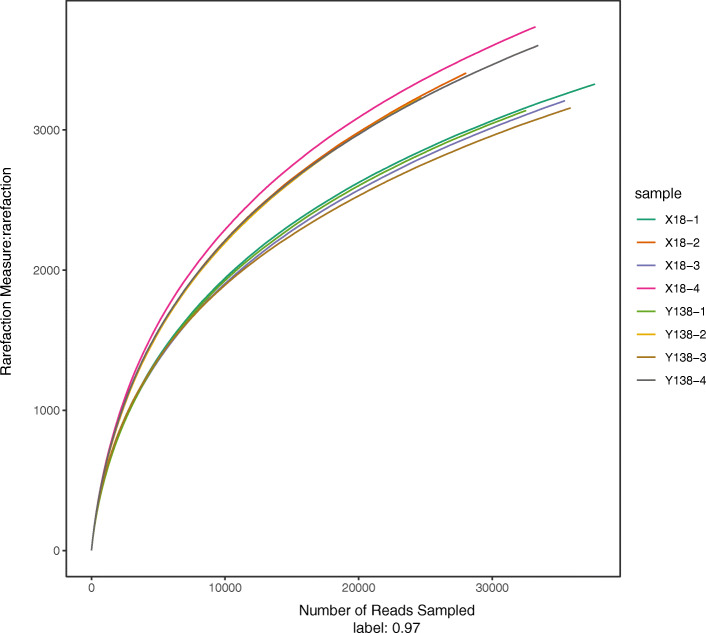
Rarefaction curves of all samples. 1 and 2 represent the early planting and pre-harvest samples in 2015; 3 and 4 represent the early planting and pre-harvest samples in 2016, respectively. The same as following

The average coverage of all samples was 96.19% (**Table** **2**). Rarefaction curves were close to plateau (**Fig.** **1**), indicating that our sequencing depth was good. The reads ranged from 24,695 – 37,688 for samples, and the operational taxonomic units (OTUs) ranged from 3,137 – 3,734. Chao, Shannon, and Simpson indexes were computed based on the bacterial OTUs. Unlike the Shannon index, the larger the Simpson value, the lower the community diversity. The Chao and Shannon values of the X18 and Y138 soils were higher in the pre-harvest period than at the beginning of planting, while the opposite was true of the Simpson index, indicating that two communities had higher species richness and diversity in the pre-harvest period.

**Table 2:** MiSeq sequencing results and α-diversity index of sweet potato rhizospheric soil samples

Sample ID	Reads	0.97				
		OTU	Chao	Coverage	Shannon	Simpson
X18-1	37688	3326	4626 (4440, 4842)	0.969407	6.69 (6.68, 6.71)	0.0033 (0.0032, 0.0034)
X18-2	28047	3405	4955 (4743, 5201)	0.954327	6.91 (6.89, 6.93)	0.0028 (0.0027, 0.0029)
X18-3	35422	3207	4509 (4324, 4724)	0.967139	6.67 (6.65, 6.68)	0.0034 (0.0033, 0.0035)
X18-4	33236	3734	5250 (5046, 5485)	0.960434	7.02 (7.01, 7.04)	0.0023 (0.0022, 0.0023)
Y138-1	32513	3137	4388 (4209, 4596)	0.964876	6.64 (6.62, 6.66)	0.0036 (0.0035, 0.0037)
Y138-2	24695	3230	4808 (4590, 5061)	0.948654	6.89 (6.87, 6.91)	0.0028 (0.0027, 0.0029)
Y138-3	35866	3156	4511 (4316, 4739)	0.968187	6.75 (6.74, 6.77)	0.0028 (0.0028, 0.0029)
Y138-4	33436	3602	5074 (4873, 5307)	0.961897	6.97 (6.95, 6.98)	0.0024 (0.0024, 0.0025)

X18: Xushu 18; Y138:Yizi 138; 1 and 2 represent sampling of early planting and early harvest in 2015, 3 and 4 represent sampling of early planting and early harvest in 2016, respectively; OTU: operational taxonomic unit; The numbers within parentheses are the lower and upper limits in statistics of the corresponding date, respectively

After X18 continuous cropping, the Chao index decreased by 2.53% and the Shannon and Simpson indices were basically unchanged at the beginning of planting in 2016 than in 2015; in the pre-harvest period, the Chao and Shannon indices increased by 5.95% and 1.59%, respectively, and the Simpson index decreased by 17.86%. After Y138 continuous cropping, the Chao and Shannon indices increased by 2.80% and 1.66%, respectively, and the Simpson indices decreased by 22.22% at the beginning of planting in 2016 than in 2015; in the pre-harvest period, the Chao and Shannon indices increased by 2.53% and 1.16%, respectively, and the Simpson index decreased by 14.29%. These indicated that the diversity and richness of rhizosphere soil bacteria of X18 and Y138 also increased after continuous cropping.

X18 had higher Chao and Shannon indices than Y138, which were contrary to the Simpson index. In other words, the bacterial richness and diversity were higher in X18 rhizospheric soil than in Y138 soil.

### Community composition analysis of rhizospheric soil bacteria

**Fig. 2:**
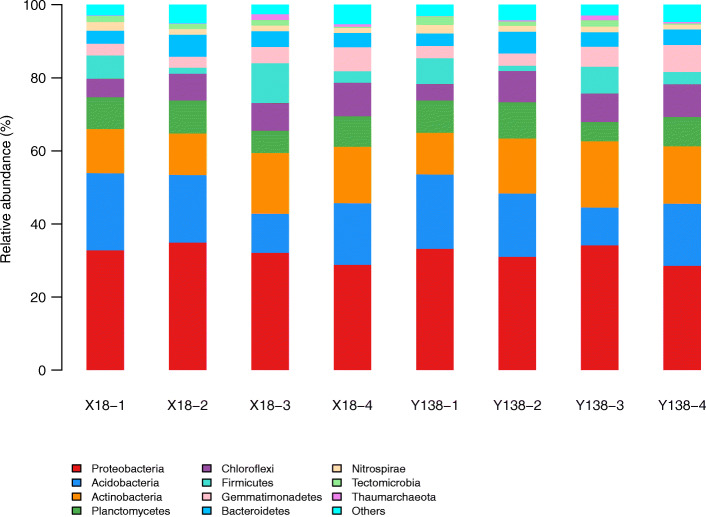
Relative abundance of rhizospheric soil bacterial phyla

At the phylum level (**Fig.** **2**), the dominant bacterial phyla of all samples were *Proteobacteria* (28.5%–34.9%), *Acidobacteria* (10.4%–21.1%), *Actinobacteria* (11.3%–18.1%), *Planctomycetes* (5.2%–9.9%), *Chloroflexi* (4.6%–9.1%), *Bacteroidetes* (3.4%–6.1%), *Gemmatimonadetes* (3.0%–7.4%), and *Firmicutes* (1.4%–10.9%). Among them, *Proteobacteria* was the most abundant phylum, followed by *Acidobacteria* and *Actinobacteria.*

After X18 and Y138 continuous cropping, *Proteobacteria* decreased by 17.30% and 8.05% , respectively, in the pre-harvest period. *Acidobacteria* showed a decreasing trend and finally increased slightly, while *Actinobacteria* showed the opposite trend. After continuous cropping, the *Firmicutes* content of X18 soil increased by 71.54% and 97.66% in the early planting and early harvest stages, respectively, and by 4.10% and 129.57%, respectively in Y138 soil. Moreover, In both varieties, the *Firmicutes* contents was higher at the beginning of planting than in the early harvest period of the same year. However, the opposite trend was observed for *Planctomycetes*. The *Planctomycetes* content of X18 in the early planting and early harvest stages decreased by 30.36% and 7.35%, respectively, while that of Y138 decreased by 40.51% and 19.04%, respectively. Further, the *Chloroflexi* and *Gemmatimonadetes* contents showed increasing trends. In X18 and Y138 rhizospheric soils, *Chloroflexi* increased by 81.09% and 96.69%, respectively ,and *Gemmatimonadetes* increased by 103.11% and 122.56%, respectively. *Gemmatimonadetes* was higher in Y138 rhizospheric soil than in X18 soil, especially in 2016.

**Fig. 3:**
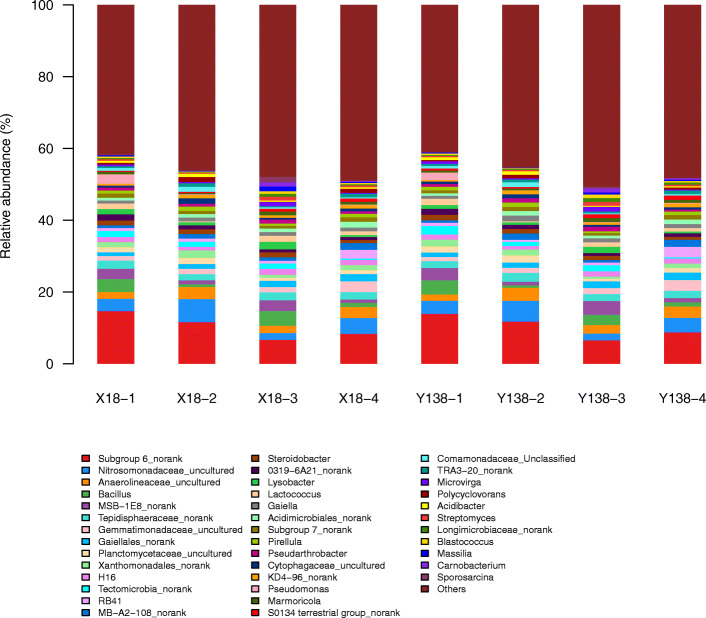
Relative abundance of rhizospheric soil bacterial genera

At the genus level (Fig. 3), *Subgroup 6_norank* (6.59% – 14.74%), *Nitrosomonadaceae_uncultured* (1.83%–6.40%), and *Anaerolineaceae_uncultured* (1.75%–3.63%) were the top three dominant bacteria genera in all rhizospheric soils of X18 and Y138. Other major genera were *Bacillus* (0.65%–4.14%), *MSB-1E8_norank* (0.87%–3.83%), *Tepidisphaeraceae_norank* (1.71%–2.56% ), *Xanthomonadales_norank* (0.62%–2.08%), and *Lysobacter* (0.55%–2.06%). After two years of continuous cropping, *Subgroup 6_norank* displayed decreasing trends in the X18 and Y138 rhizospheric soils, decreasing by 54.34% and 52.66%, respectively, and then increased slightly in the 2016 pre-harvest period. However, *Nitrosomonadaceae-uncultured* and *Anaerolineaceae-uncultured* were present at low levels at the beginning of planting, then were increased in the pre-harvest period of the same year. *Bacillus* and *Lysobacter* showed the opposite trend. Moreover, in every sampling period, *Lysobacter* was higher in the rhizospheric soil of X18 than in that of Y138. This was also true of *Bacillus,* except for the beginning of planting in 2015. Furthermore, in2016, the reduction of *Lysobacter* in X18 and Y138 rhizosphere soil was 1.3 and 2.4 times greater than in 2015.

### Venn analysis of rhizospheric soil bacteria

**Fig. 4:**
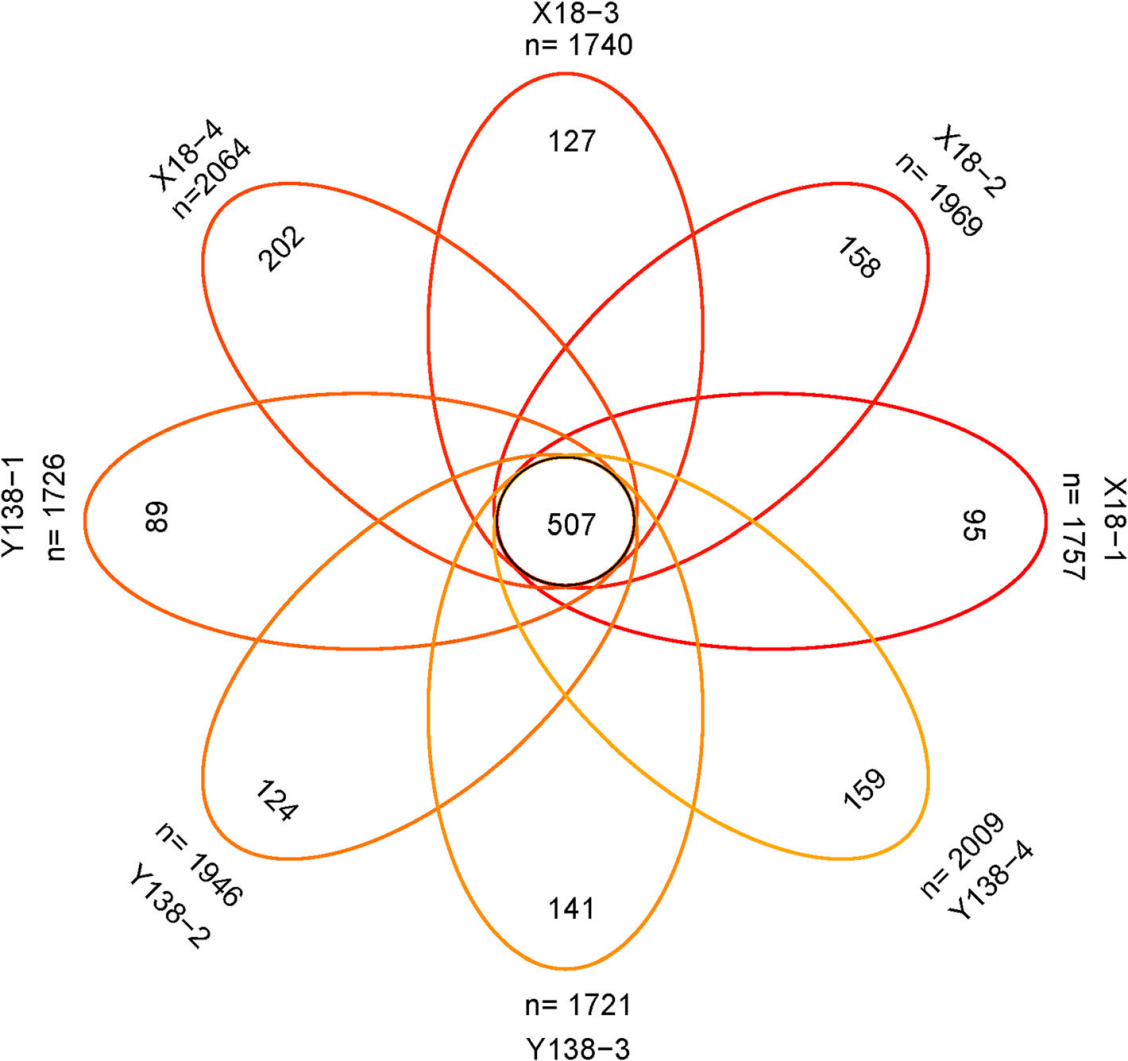
Number of common and unique OTUs based on Venn analysis

Venn diagram revealed the overlapped and unique OTUs of all samples (Fig. 4). After two years of continuous cropping, the OTUs shared by all samples was 507. In the four sampling periods, there were 95, 158, 127, and 202 unique OTUs in the rhizospheric soils of X18, and 89, 124,141, and 159 unique OTUs in the rhizospheric soils of Y138 . As continuous cropping year increased, the specific OTUs in X18 soil increased by 33.68% and 27.85% in the early planting and early harvest periods, while the OTUs in Y138 soil increased by 58.43% and 28.23% in these periods.

The number of OTUs specific to X18 was greater than that specific to Y138 (except for the beginning of planting in 2016), indicating that continuous cropping led to changes in the bacterial communities in X18 and Y138 rhizosphere soil. These differences were largest during the early harvest period of 2016.

### Heatmap, clustering, and principal component analysis (PCA) of rhizospheric soil bacteria

**Fig. 5:**
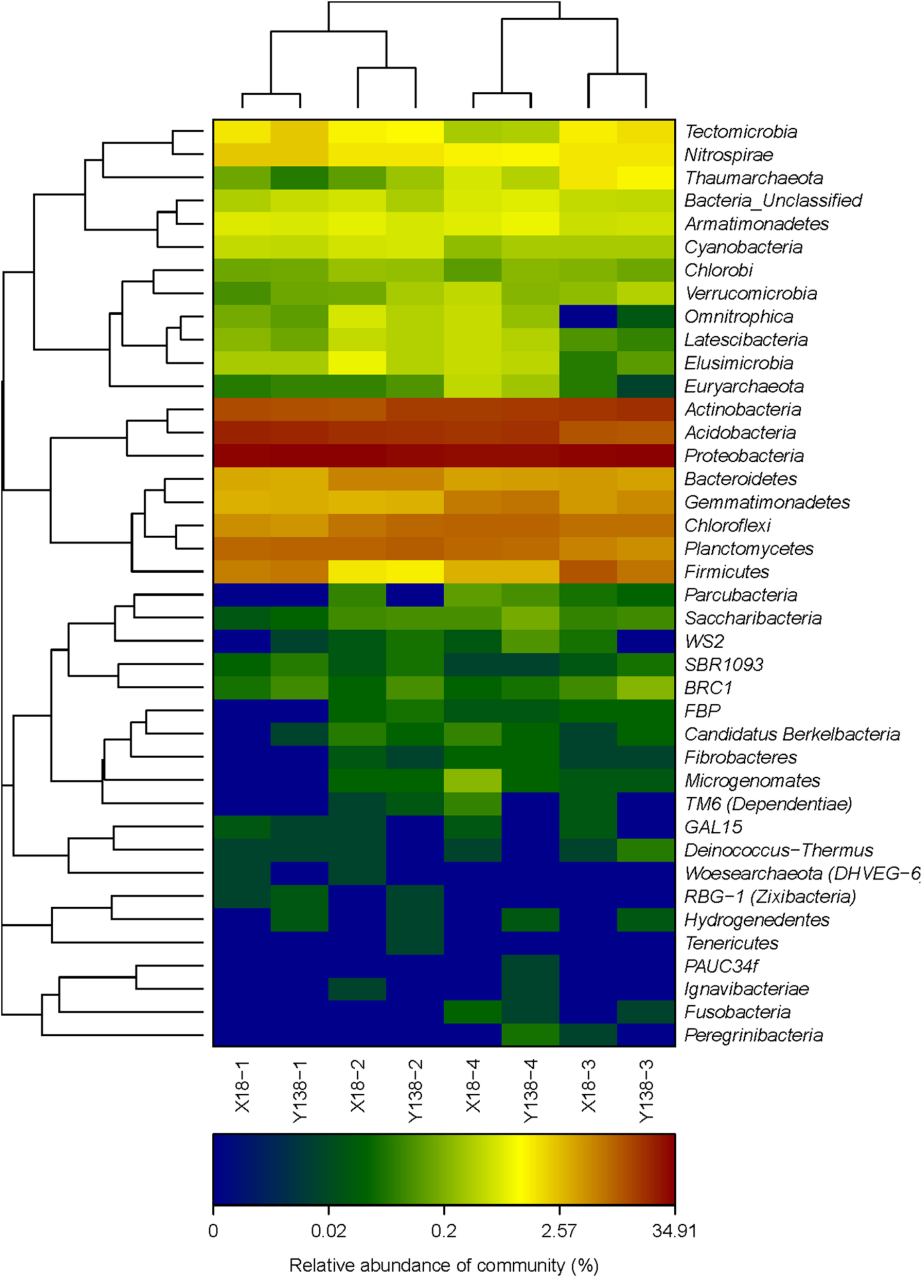
Microbial community heatmap and cluster analysis of the bacterial phyla

The results of heatmap and clustering analysis of 40 phyla in all samples are illustrated in Fig. 5, and clearly demonstrate the differences in rhizospheric soil bacterial composition between the X18 and Y138 varieties. The samples grouped into two clusters and samples from the same consecutive cropping times clustered together. In addition, X18 and Y138 samples from the same sampling periods grouped together.

**Fig. 6:**
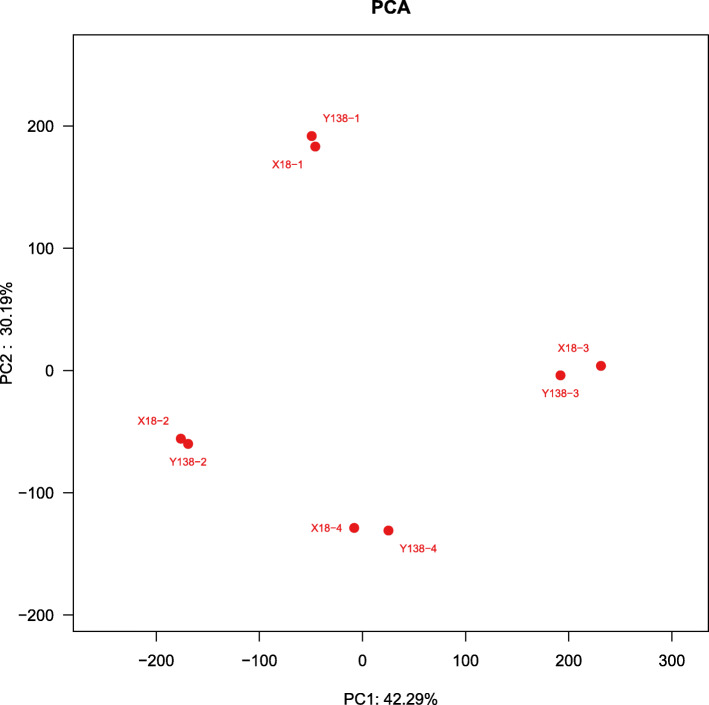
Principal component analysis (PCA) of OTUs

The OTUs of X18 and Y138 were subjected to PCA. The extracted two principal components explained 72.48% of the total variation (Fig. 6). As continuous cropping time increased, samples from different sampling times became farther apart. However, at the same sampling times, X18 and Y138 samples were relatively close to each other. With continuous cropping, the distance between these samples also gradually increased, which indicated that differences between their bacterial communities were also increasing. These results were consistent with the results of the heatmap and cluster analyses in Fig. 5. Overall, the results suggested that (i) continuous cropping led to bacterial community structure changes in X18 and Y138 rhizosphere soil; (ii) rhizospheric soil bacterial community structures of X18 and Y138 were similar in the same sampling period.

### Relationship between bacterial phyla and physicochemical characteristics of sweet potato rhizospheric soil

**Fig. 7:**
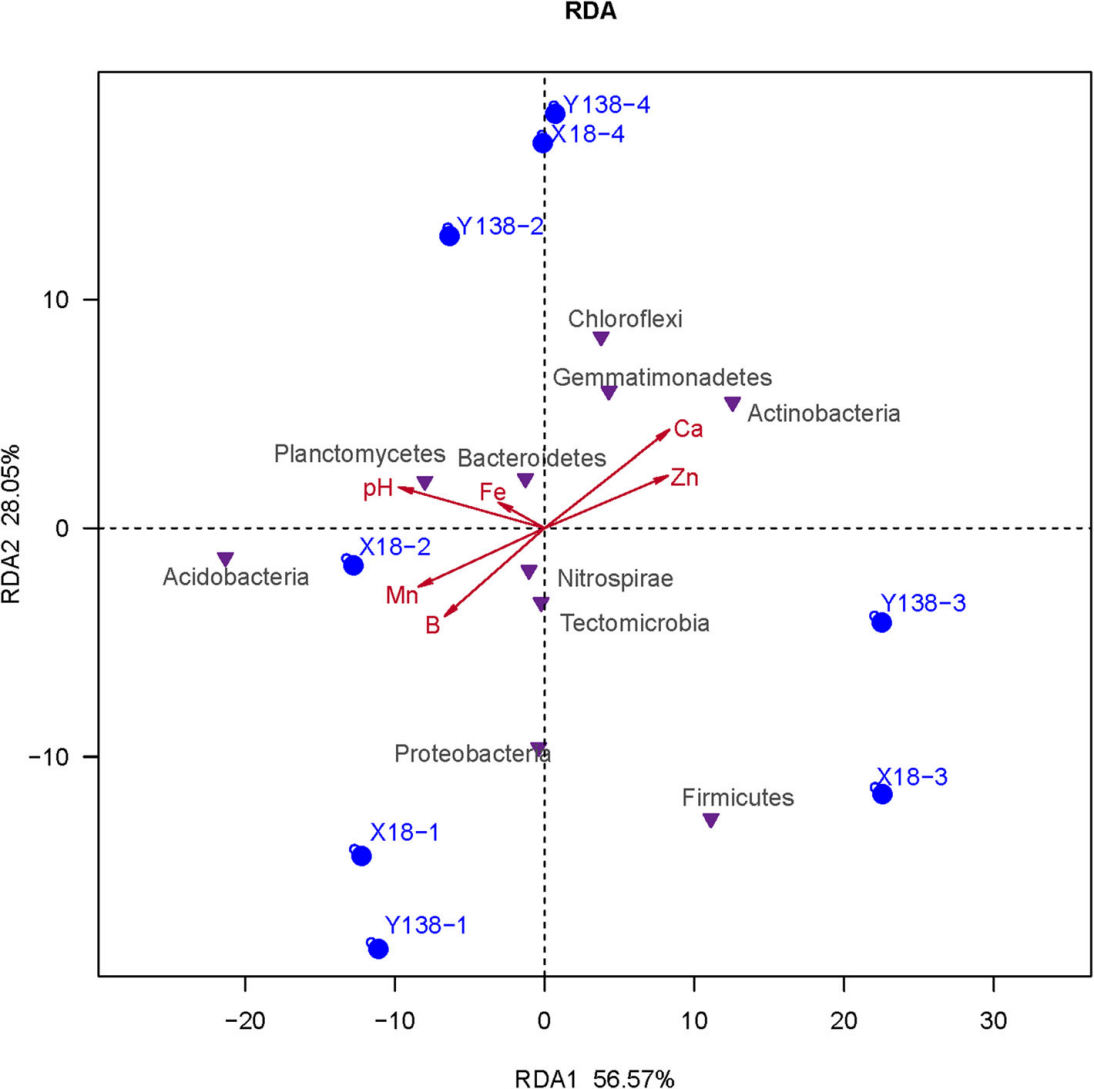
Redundancy analysis of the 10 dominant bacterial phyla and soil physicochemical properties. Ca: available calcium; B: available boron; Fe: available iron; Mn: available manganese; Zn: available zinc. The same as following

The results of redundancy analysis (RDA) on top ten bacterial phyla and environmental factors of X18 and Y138 rhizospheric soil are showed in Fig. 7. RDA1 and RDA2 explained 56.57% and 28.05% of the total variation, respectively. The magnitude of the effects of soil properties on bacterial community structure had the following order, soil pH > Ca > Mn > Zn > B > Fe. The results showed that soil pH (r^2^=0.9737, Pr=0.004) and available Ca (r^2^=0.8815, Pr=0.011) were significantly correlated with the bacterial community. It indicated that pH was a strong predictor of the X18 and Y138 rhizospheric soil bacterial community compositions.

**Fig. 8:**
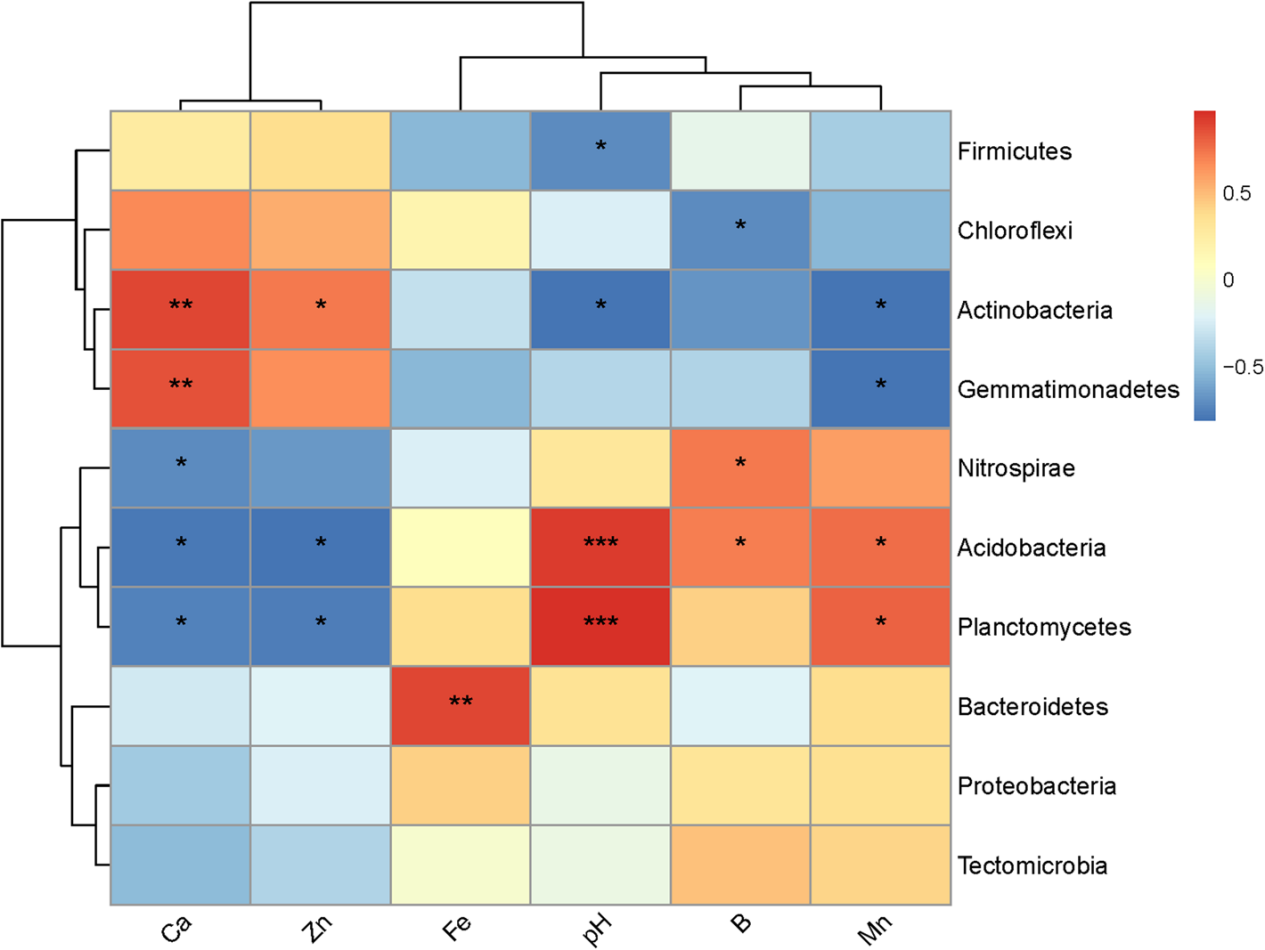
Correlations between the 10 dominant bacterial phyla and soil properties. *, P<0.05; **, P<0.01; ***, P<0.001

In addition, the results of Spearman’s correlation coefficient analysis were as follows (Fig. 8). pH was positively correlated with *Planctomycetes* (R=0.97) and *Acidobacteria* (R=0.93), but had negative correlations with *Actinobacteria* (R = −0.79) and *Firmicutes* (R = −0.72); available Ca was positively related to *Actinobacteria* (R=0.89) and *Gemmatimonadetes* (R=0.86), and was inversely correlated with *Acidobacteria* (R = −0.79), *Planctomycetes* (R= −0.75), and *Nitrospirae* (R= −0.72). At the same time, it can be seen from Fig. 8 that the soil physicochemical properties were divided into two groups, with available Ca and available Zn clustered into one group and the rest clustered into another, indicating that available Ca and available Zn had similar effects on the bacteria, which were different from the rest.

## Discussion

To obtain a more comprehensive understanding of the rhizospheric soil bacterial community structure of sweet potatoes, the Illumina MiSeq method was used in this study [33, 34]. The V4–V5 highly variable region was selected as the sequencing region because a previous study demonstrated that this region was the best sequencing region among nine highly–variable regions [35].

We found that the Shannon index increased with continuous cropping, indicating an increase in bacterial diversity in the rhizospheric soil. This was aligned with previous studies on *Andrographis paniculata* and potatoes [36, 37], but contrary to *Sophora flavescens* and *Lycium barbarum* L. under continuous cropping conditions [29, 30]. The differences may be due to variations in the duration of continuous cropping, soil environmental conditions, plant types, etc.

The most dominant bacterial phylum in the X18 and Y138 rhizospheric soil was *Proteobacteria*, which was in accordance with published reports [8, 24, 36, 38]. The main function of *Proteobacteria* is to decompose organic matter and promote plant growth [39, 40]. The most dominant genus was *Subgroup 6_norank*, which accorded with Yin et al. [24]. As continuous cropping time increased, the *Proteobacteria* content decreased, which was in agreement with Liu et al. [41], while was inconsistent with the report on *A. paniculata* [36]. *Acidobacteria* showed a tendency to decrease gradually and then increase slightly. *Actinobacteria* tended to increase gradually and then decrease slightly, which was almost consistent with Yin et al. [24] and our previous study [42], but differed from the results of Li et al. [8] and Wu et al. [27]. These discrepancies might be due to differences in plant species, plant genotypes, continuous cropping durations, and soil types, which also cause different changes in soil microorganisms [14, 43–45].

We also found that some microbial changes were affected by the sweet potato growth stage. For instance, the content of *Firmicutes* was high at the beginning of planting and decreased in pre-harvest period. This indicates that the growth of sweet potato has a certain influence on it. After the sweet potatoes were harvested, its content recovered. These changes may also be related to the season. Li et al. [46] found that seasonal changes have important impacts on soil microbes, but that different microbes vary with season differently.

As continuous cropping time increased, the content of *Lysobacter* decreased. It is well known that *Lysobacter* is an important biocontrol bacteria with strong bacteriolytic and bacteriostatic effects [47]. For example, *Lysobacter enzymogenes* OH 11 was found to exert a significant bacteriostatic effect on sweet potato soft rot pathogen. Conversely, *Gemmatimonadetes*, a harmful bacteria that can lead to N loss and reduce crop growth [40], increased with continuous cropping.

Sweet potato continuous cropping changed the bacterial community structure, which was aligned with previous reports [8, 26, 27]. These changes further increased the continuous cropping obstacles of X18 and Y138**.** The average yields of the two varieties both decreased, X18 decreased from 12.36 to 10.64 t hm^**-2**^, and Y138 decreased from 9.15 to 2.66 t hm^-2^.

Consequently, maintaining a balance of bacterial community structure is very important for prevention and control continuous cropping obstacles. In practice, microbial fertilizers and soil modifiers [48–50] can be applied to maintain the microecological balance.

We observed differences in rhizospheric soil bacterial community composition between the two sweet potato varieties after continuous cropping. The number of OTUs specific to X18 increased more than those specific to Y138, and the community structures became increasingly different. According to α-diversity analysis, the rhizospheric soil of X18 had higher bacterial richness and diversity. Thus, the community structure of X18 was relatively stable, its tolerance to soil environmental changes was greater, and its resistance to continuous cropping obstacles was stronger, while the community structure of Y138 was more susceptible to continuous cropping. Moreover, *Lysobacter* and *Bacillus* were higher in the X18 rhizosphere soil than in the Y138 soil. Many studies have shown that *Bacillus* may resist some soil–borne diseases [14, 27, 40]. Higher *Lysobacter* and *Bacillus* contents would therefore be more conducive to the growth of X18. In contrast, the rhizospheric soil of Y138 contained more harmful *Gemmatimonadetes*. Therefore, it is also important to choose varieties of sweet potato that are resistant to continuous cropping in production practice.

After sweet potato continuous cropping, rhizospheric soil physicochemical properties were also changed. Moreover, it was found that pH had the greatest impact on the bacterial community structure, which was consistent with several published reports [8, 51, 52]. Soil pH can affect soil microbial physiological metabolism, alter competitive relationships within microbial communities, and inhibit the growth of non-adapted microbes [29], all of which affect microbial community structure. In terms of the three bacterial phyla with the highest relative content, *Acidobacteria* was positively correlated with pH, which was in line with Yang et al. [40]; *Actinobacteria* was negatively correlated with pH, which was in agreement with previous studies [27, 53]. However, the results were different from those reported by Li et al. [8], which might have been due to difference in plant age or genotype [54].

Similar to bacteria, soil fungi are important decomposers. However, some fungi are strongly associated with plant diseases [55, 56]. The results of our previous study indicated that fungal diversity and abundance in rhizospheric soil were significantly increased after continuous cropping. Furthermore, the contents of *Fusarium*, *Verticillium*, and *Colletotrichum,* the pathogens of sweet potatoes, were increased. These changes affected the balance of the fungal community structure [7].

Overall, after continuous cropping, the microbial community structure of sweet potato rhizospheric soil was changed, which might be a crucial aspect contributing to continuous cropping obstacles. Thus, maintaining the microecosystem balance of the rhizosphere soil is crucial to relieve the continuous cropping obstacles. In addition, continuous cropping obstacles are related to many factors. It is necessary to combine with many other methods to solve this problem. Therefore, we will perform further in-depth research on the mechanisms of sweet potatoes continuous cropping obstacles.

## Conclusions

The continuous cropping of sweet potatoes obviously affected the bacterial community structure, physical and chemical properties of the rhizosphere soil. The bacterial diversity increased; however, the changes in the soils of two sweet potato varieties were different. The contents of *Lysobacter* and *Bacillus* were higher in the sweet potato variety that was resistant to continuous cropping. It provides a basis for the development of specialized microbial fertilizers for sweet potatoes. Therefore, further research is needed to see if *Lysobacter* and *Bacillus* could be used to produce microbial fertilizers to mitigate continuous cropping obstacles of sweet potatoes.

## Methods

### Study area and materials

Our study was conducted at the Dishang test station of Institute of Cereal and Oil Grops, Hebei Academy of Agriculture and Forestry Sciences, China (37°56′24.62″ N, 114°42′46.96″ E) [7]. There was a warm temperate sub - humid continental monsoon climate and the local soil type is Mottlic Hapli-Ustic Argosols (cinnamon soil). Two sweet potato varieties were used as experimental materials, one was X18 that was resistant to continuous cropping and the other was Y138 that was susceptible to continuous cropping. Both varieties were collected from the Institute of Cereal and Oil Crops, Hebei Academy of Agriculture and Forestry Sciences.

### Experimental design and sample collection

Sweet potatoes were planted in May and harvested in October each year, and were continuously planted for 2 years (2015–2016). After each harvest, the fields were left fallow for the rest of the year. A random block design was used, with three experimental replicates. Details of the plot can be found in our previous report [7]. Agronomic management was the same for all fields over the two years. Rhizospheric soil was collected 30 days after planting and 7 days before the harvest, for a total of four sampling periods. Rhizospheric soil samples were taken by the method of Sun et al. [57]. For each sweet potato variety at each sampling point, the rhizospheric soils of 15 sweet potatoes located in an “S” shape were collected from each plot and mixed thoroughly to generate one composite soil sample. All samples were quickly taken to the laboratory in a cooler. Each sample was filtered through a 2-mm sieve, and then divided into two portions. One portion was stored at −80°C for DNA analysis, the other portion was air-dried for soil characteristics analysis.

### Soil physicochemical property analysis

Soil pH was determined by the electrode method (2.5:1 water: soil ratio) [40], and available Ca, B, Fe, Mn, and Zn were measured using the atomic absorption method according to Du and Gao [58].

### DNA extraction, amplification, and sequencing

The DNA was extracted with an E.Z.N.A. ® Soil DNA Kit following the instructions. The V4–V5 regions were amplified by polymerase chain reaction (PCR) with 515F 5′-barcode-GTGCCAGCMGCCGCGG)-3′ and 907R 5′-CCGTCAATTCMTTTRAGTTT-3′, where barcode was an 8-base sequence unique to each sample. PCR reactions and amplicons extraction and purification were performed by the method of Yang et al. [40]. Purified PCR products were quantified using Qubit®3.0, and every 24 amplicons with different barcodes were equally mixed. Illumina Pair-End library construction and sequencing were performed according to our previous report [7]. Complete data sets were deposited in the NCBI Sequence Read Archive database under accession number SRP214716.

### Processing and statistical analysis of sequencing data

To remove low quality sequences, quality control and sequence quality filtering were applied according to Yang et al. [40]. OTUs were clustered with 97% similarity cutoff with UPARSE (version 7.1 http://drive5.com/uparse/) and chimeric sequences were identified and removed with UCHIME. RDP Classifier was used to analyze the phylogenetic affiliations of each 16S rRNA gene sequence against the SILVA (http://www.arb-silva.de) database with a confidence threshold of 70% [40].

Chao, Shannon, and Simpson indexes were calculated with Mothur v.1.21.1 [59]. PCA was performed with R-forge (the PCA graphics was generated with Vegan 2.0 package). Shared and unique OTUs were analyzed using Venn diagrams by VennDiagram. RDA was performed to analyze the correlations between environmental factors and bacterial community composition. Spearman’s rank correlations coefficients and Heatmap figures were performed with Vegan packages in R. The data of the physicochemical properties of rhizospheric soil were analysed by one-way analysis of variance (ANOVA) (P < 0.05) in SPSS v21.0 [40].

## Data Availability

The datasets generated and analysed during the current study are available in the NCBI Sequence Read Archive repository under accession number SRP214716 (https://submit.ncbi.nlm.nih.gov/subs/sra/SUB7627087/overview).

## Abbreviations

X18: Xushu18
Y138: Yizi138
OTUs: Operational taxonomic units
PCA: Principal component analysis
RDA: Redundancy analysis
ANOVA: One-way analysis of variance
