# Correction to: Effects of continuous cropping of sweet potatoes on the bacterial community structure in rhizospheric soil

**DOI:** 10.1186/s12866-021-02194-2

**Published:** 2021-04-20

**Authors:** Zhiyuan Gao, Yaya Hu, Meikun Han, Junjie Xu, Xue Wang, Lanfu Liu, Zhonghou Tang, Weijing Jiao, Rong Jin, Ming Liu, Zhengjun Guan, Zhimin Ma

**Affiliations:** 1 Institute of Cereal and Oil Crops, Hebei Academy of Agriculture and Forestry Sciences, The Key Laboratory of Crop Genetics and Breeding of Hebei, Shijiazhuang, China; 2 Agricultural Product Quality Inspection Center of Shijiazhuang, Shijiazhuang, China; 3 Xuzhou Sweet Potato Research Center, Xuzhou Institute of Agricultural Sciences, Xuzhou, China; 4 Department of Life Science, Yuncheng University, Yuncheng, China

**Correction to: BMC Microbiol 21, 102 (2021)**

**https://doi.org/10.1186/s12866-021-02120-6**

Following the publication of the original article [1], we were notified that author affiliations 2 and 4 have been swapped by mistake by the authors. They should actually be:

**2** Agricultural Product Quality Inspection Center of Shijiazhuang, Shijiazhuang, China.

**4** Department of Life Science, Yuncheng University, Yuncheng, China.

The original article has been corrected.
